# Dietary-Nutraceutical Properties of Oat Protein and Peptides

**DOI:** 10.3389/fnut.2022.950400

**Published:** 2022-07-05

**Authors:** Hamad Rafique, Rui Dong, Xiaolong Wang, Aamina Alim, Rana Muhammad Aadil, Lu Li, Liang Zou, Xinzhong Hu

**Affiliations:** ^1^College of Food Engineering and Nutritional Science, Shaanxi Normal University, Xi’an, China; ^2^National Institute of Food Science and Technology, University of Agriculture, Faisalabad, Pakistan; ^3^Guilin Seamild Food Co., Ltd., Guilin, China; ^4^School of Food and Biological Engineering, Chengdu University, Chengdu, China

**Keywords:** oat protein, bioactive peptides, antioxidant, antidiabetic, antihypertensive, immunomodulatory, antifatigue, antihypoxic

## Abstract

Oats are considered the healthiest grain due to their high content of phytochemicals, dietary fibers, and protein. In recent years, oat protein and peptides have gained popularity as possible therapeutic or nutraceutical candidates. Generally, oat peptides with bioactive properties can be obtained by the enzymatic hydrolysis of proteins and are known to have a variety of regulatory functions. This review article focused on the nutraceutical worth of oat proteins and peptides and also describes the application of oat protein as a functional ingredient. Outcomes of this study indicated that oat protein and peptides present various therapeutical properties, including antidiabetic, antioxidant, antihypoxic, antihypertensive, antithrombotic, antifatigue, immunomodulatory, and hypocholestrolaemic. However, most of the conducted studies are limited to *in vitro* conditions and less data is available on assessing the effectiveness of the oat peptides *in vivo*. Future efforts should be directed at performing systematic animal studies; in addition, clinical trials also need to be conducted to fully support the development of functional food products, nutraceutical, and therapeutical applications.

## Introduction

Oats are one of the most nutritious grains and are the 5th most consumed crop with an annual production of 23 million tons globally (1, 2). Generally, oat grains can be classified as naked oats (*Avena nuda* L.) and hulled oats (*Avena stiva* L.). Oats contain a relatively high content of protein, and it also comprises a considerable amount of lipids and other bioactive compounds, such as beta-glucans, phenolics (trace–150 mg/kg), avenanthramides (26–150 mg/kg), and flavonoids 1.77 mmol/g (3, 4). Depending on different varieties, oat grain comprises lipids 5–10%, crude protein 12–20%, crude fiber 3–14%, and carbohydrates 69–76% (5, 6). The protein content of oat grain is higher as compared to other cereals, including rice 7–10%, wheat 11–15%, and millets 7–11%, while lower than legumes, such as pea 23–31% and soy 36–40%. The protein fractions and their molecular weights are also significantly different among grains as given in Table 1 (6–10).

**TABLE 1:** Protein content and molecular weight distribution of oat and other grains.

Grains	Total protein	Globulins		Albumins		Prolamins		Glutenins	
		%	MW	%	MW	%	MW	%	MW
Oat	12–20%	70–80	54–60 kDa	1–12	19–21 kDa	4–15	20–40 kDa	≤10	10–90 kDa
Wheat	11–15%	*A/G 20–25	98–100 kDa	*A/G 20–25	15 kDa	30–40	35 kDa	45	10 million kDa
Rice	7–10%	7–17	23–105 kDa	5–10	10–200 kDa	3–6	10–32 kDa	75–81	51–57 kDa
Millet	7–11%	*A/G 11–17	13–32 kDa	*A/G 11–17	13–32 kDa	*6.8–9.3 P like; 7.5–11.6	12–35 kDa	*39–54 G like; 5.9–9	10.5–56 kDa
Pea	23–31%	2.47	10–43 kDa	7.01	11–21 kDa	1.52	–	87.47	12–66 kDa

*A/G, Albumin and Globulin combine; P like, Prolamine like fraction; G like, Glutenins like fraction (11, 14–17).

Globulin is the major storage protein in oats. Mainly, oat protein is composed of four fractions; globulin 70–80%, albumins 1–12%, prolamins 4–15%, and glutenins ≤ 10% (18). The salt soluble globulin fraction is divided into 3 subunits, 12S, 7S, and 3S. The molecular weight of globulin ranges from 54 to 60 kDa, which is approximate to the 11S globulin (glycinin) structure of soy protein (12, 13); besides this, 12S has acidic A and basic B polypeptides subunits with a molecular weight of 32 and 22 kDa, respectively (19). Other subunits of globulins, 7S has polypeptides of molecular weight of 55–65 kDa, and 3S contains two polypeptides of 15 and 21 kDa (17, 18). Water-soluble albumin plays a role as an enzyme in the protein and defensive system of plants, and its molecular weight ranges from 19 to 21 kDa (20). Prolamin (avenins), the alcohol-soluble protein fraction with a molecular weight of 20–40 kDa, has structural similarities with Sulfur rich sub-units of wheat (α and γ gliadins), γ-secalins of rye, and B-hordeins of barley proteins fractions (12, 19). This protein fraction is limiting in proline and glutamine amino acids but has the same function as the storage protein fraction (gluten) of wheat (21). The minor fraction (Glutelins) comprises polypeptides having a molecular weight of 10–90 kDa (20).

Oat protein contains a comparatively higher amount of essential amino acids, especially (lysine, valine, isoleucine, threonine, histidine, and methionine) than other grains. Amino acids composition of oat protein significantly differs even among its fractions, globulins carry the highest amount of essential amino acids, such as lysine, valine, phenylalanine, and histidine, as well as non-essential amino acids, including arginine and glutamic, as compared to all three other fractions (20). In addition, oat amino acids composition meets the Food and Agriculture Organization’s recommended nutritional needs of an adult except for methionine, which is still the limiting amino acid in oat protein (22). Furthermore, the consumer’s acceptability score of oat protein is also higher than any other plant-based pea, lupin, and soy proteins (20, 23, 24).

Plant protein has received much attention in recent years, as consumers are pursuing more plant-based diets in hopes of improving health and preserving the environment. Livestock puts much higher pressure on global warming, resource, and land use than plant-based food (25). Animal-based foods, including dairy, eggs, meat, and aquaculture, use 83% of the world’s cultivated land and contribute up to 58% of food emissions, while providing only 37% of the total proteins (26). The higher environmental impact of animal products is due to the high feed consumption per kilogram of food produced, in addition, a large part of greenhouse gas emissions comes from intestinal fermentation and fecal management in ruminants (26, 27). There is a need to reduce greenhouse gas emissions by minimizing the animal-based proteins, and substituting even only a part of animal protein intake with plant-based proteins would benefit both human health and the environment (28). Numerous studies have shown that plant products have less impact on climate and eutrophication and lower land use as compared to animal-based products. For example, beans and peas have the lowest land use per kg of protein as compared to milk, eggs, and poultry (29–31). Specifically, the carbon footprint of oat protein concentrate (OPC) is more than 50% lower when compared with dairy proteins (32). OPC-enriched food products could reduce 13% of greenhouse gas emissions and 26% of land use when substituted with animal-based proteins (33); similarly, oat drinks emitted 16–41% lesser greenhouse gas as compared to cow milk (34). The use of plant proteins has considerable potential in mitigating climate change and reducing land use. From this perspective, the above-mentioned studies reveal the potential that the oat protein made it possible to substitute some animal proteins with plant proteins.

**FIGURE 1:**
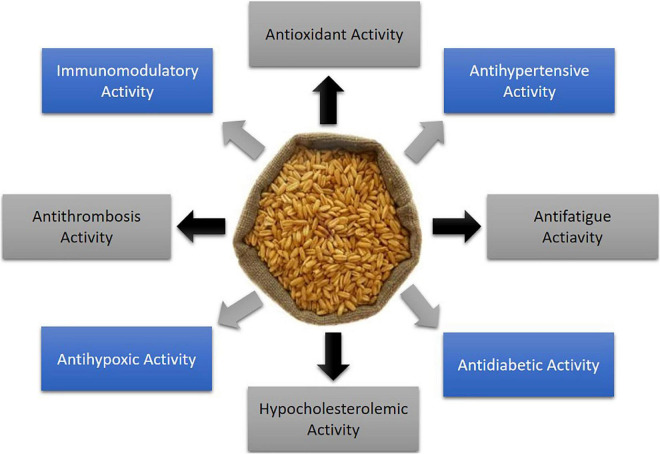
Health protecting properties of oat derived bioactive peptides.

Being of high protein content, sustainability, as well as good nutritional profile, oats are considered the promising cereals for extracting plant-based proteins and isolating bioactive peptides (35). Plant bioactive peptides, in general, are small fragments of proteins, composed of 2–20 amino acids and having less than 3 kDa molecular weight (36, 37). These peptides naturally exist or are derived from precursor proteins through gestational simulation, microbial fermentation, or enzymatic hydrolysis (38). It has been found that bioactive peptides have a simpler structure, higher stability, and more remarkable physiological activities and functions compared to protein (39). In this way, oat protein-derived bioactive peptides have been identified to exert distinct health improving properties, such as antidiabetic, immunomodulatory, antifatigue, antithrombosis, antihypoxic, antihypertensive, hypocholestrolaemic, and antioxidant effects (Figure 1). In this article, we mainly focus on the oat proteins in detail for their application as a functional ingredient and potential nutraceutical activities (Table 2).

**TABLE 2:** Oat protein derived hydrolysates/peptides and their bioactivity.

Protein fraction	Protease	Hydrolysates/peptides	Bio activity	References
Total protein	Alcalase Flavourzyme Papain, protamex	Hydrolysates	Exhibited the hydroxyl, peroxyl, ABTS radical scavenging, and Fe 2^+^ chelating activities *in vitro*	(53)
Globulin protein	Alcalase	IRIPIL, FLKPMT, NSKNFPTL, LIGRPIIY, FNDILRRGQLL	Exhibited DPPH and hydroxyl radical scavenging activity *in vitro*	(54)
Total protein	Peptides Synthesized by GenScript	FNDRLRQGQLL, GLVYIL, GQTV, GQTVFNDRLRQGQLL, YHNAP, YHNAPGLVYIL, DVNNNANQLEPR	Displayed the Peroxyl radical scavenging (ORAC) and cytoprotective capacity in stressed HepG2 hepatic cells	(55)
Total protein	Flavourzyme Papain Alcalase	Hydrolysates	Showed Peroxyl (ROO.), superoxide (O.), and hydroxyl (HO.) radical scavenging activity *in vitro*	(63)
Total protein	Alcalase, Papain, Flavourzyme, Protamex	Hydrolysates, pretreated with cellulose degrading enzymes	Improved the activities of Antioxidant enzymes, including CAT, SOD, and GPx, in stressed induced hepatic cells	(56)
Oat bran	–	Oat peptide-ferrous (OP-Fe^+2^) chelate	Alleviated the oxidative by increasing the activity of SOD and GSH and down-regulating MDA content in rats	(58)
Oat bran extract		Oat peptides	Reversed the H_2_O_2_ induced decrease of superoxide Dismutase and inhibited malondialdehyde in Human dermal fibroblast	(59)
Total protein	Papain	YFDEQNEQFR, GQLLIVPQ, SPFWNINAH, NINAHSVVY, RALPIDVL	Inhibited the lipid oxidation and linoleic acid peroxidation also inhibited the α-amylase *in vitro*	(57)
Globulin protein	Trypsin	Hydrolysates And LQAFEPLR, EFLLAGNNK	Competitively suppressed the DPP4 and alfa-glucosidase downregulated the protein expression of DPP4, while elevated the protein expression of α-glucosidase, GLUT2 and GLUT 4 in Caco-2 cell lines	(61)
Total protein	Flavourzyme Papain,Alcalase	Hydrolysates	Inhibited the dipeptidyl peptidase-4 and α-amylase. Improved the secretion of glucagon like peptide-1 in NCI-H716 cell lines	(63)
Total protein	Chemically synthesized	Protein, FFG, IFFFL, PFL, WCY, YPIL, CPA, FLLA, and FEPL	Inhibited the secretion of Angiotensin-1 converting enzyme and Renin *in vitro*	(70)
Globulin	Alcalase, Flavourzyme, Pepsin, Trypsin	SSYYPFK selected based on *in silico* analysis	Inhibited the activity of Angiotensin-1 converting enzyme and Renin and ET-1 *in vitro*	(72)
Total protein	Multiple proteases	Mixture of Oligo-peptides	Improved innate and adaptive immunity *via* regulating Cytokine’s secretion, antibody production and T cells stimulation in rat’s model	(75)
Total protein	–	Oligopeptides	Improved the hypoxia by regulating LDH, MDA, HB, HCT, RBC, VEGF, and mRNA expression in rats’ model	(81)
Total protein	–	Oatmeal, protein isolates	Effectively improved the fatigue by increasing liver glycogen, SOD, LDH, and reducing the BUN and MDA in rats’ model	(88)
Total protein	–	Oat protein isolates	Alleviated the exercise induced fatigue by reducing plasma myoglobin, IL-6, creatinine kinase, and C reactive protein content. Also inhibited the limb edema following damaging exercise and lessened the adverse effects on muscle strength in human clinical trial	(95)
Total and globulin protein	Pepsin Pencreatin and trypsin	Hydrolysates and peptides	Inhibited the Arachidonic acid induced platelet aggregation by acting on COX1-TXA_2_ synthase pathway to produce TXA_2_ *in vitro*	(100)
Total Protein	–	Protein isolates	Increased the excretion of total cholesterol and bile acids, consequently decreased in plasma level of low-density lipoprotein, liver total cholesterol and activity of liver 7α-hydroxylase (CYP7A1) increased in animal model	(103)

## Application of Oat Protein as a Functional Ingredient

Interest in protein-rich diets is increasingly favored by their health-promoting effects, with a special focus on sources. Plant-based protein is replacing animal protein as it is considered better for human consumption, as well as safe for the planet (40). The market request for developing plant protein-based products is continuously increasing because of high concern toward health, animal welfare, and environmental protection. This searching desire for alternative non-animal protein sources opens the way to the valorization of non-fully exploited plants and industrial by-products (33). With the increase in demand for nutrition products, oat protein-infused beverages, bakery items, supplements, and others are making their way into the market, as it contains high-quality protein and is also suitable for flavoring. Numerous studies have been conducted to review the possibility of using oat for the development of nutraceuticals. The lipid-lowering and antioxidant properties of oat protein make it a potential ingredient for nutraceuticals (41). Oat protein (OP) gels have been used for the preparation of prebiotic loaded nutraceuticals which can prevent the deterioration of prebiotics in gastric conditions. OP gels have been shown to resist pepsin digestion and have the capacity to release bioactive compounds during gastrointestinal simulation conditions (19). Due to growing interest in plant protein ingredients, oat has gained much attention for its unique amino acid composition, protein quality, and protein content. The global oat protein market reached US$ 48 million in 2018 and is expected to grow up to US$ 63 million by the end of 2025 with a compound annual growth rate of 4.1% during 2019–2025 (42). Regarding usage and composition, oat protein increased from 884t in 2012 to 1398 t in 2017 globally, with a compound annual growth rate of more than 9.6% (43).

In terms of using oat protein in food applications, oat protein has better sensory properties than legume and oilseed proteins (44). Oat protein concentrates and isolates can be easily incorporated into pasta and bakery products to improve their protein content. Although well suited to bakery products, the applicability of oat protein is still limited in semisolid and liquid foods because of its low solubility in natural and mildly acidic conditions (45). Oat protein is also a good replacer of skim milk powder because of its ability to produce better quality yogurt in terms of mouthfeel and syneresis. OPC due to the excellent techno-functional properties of the contained oat starch is an appropriate ingredient for the implementation of the nutritional value of oat protein, giving a healthier and more natural shape to the yogurt (46). Given the increased demand for a plant protein source, meat analogs produced from plant proteins have gained traction (47). The meat analogs’ market has long been dominated by pea, wheat gluten, and soy proteins (28). However, due to the presence of common allergens in pea and soy protein, the oat protein has the potential to play a significant role in the meat alternative market as a new protein ingredient (48). Besides, using oat protein in meat alternative analog or yogurt, oat milk is also included in the list of common oat products that dominate the market. The oat milk is a promising substitute for dairy products because it can be used in combination with probiotics to prepare fermented products like yogurt. Due to consumer awareness about plant-based milk and increased interest in flexitarian, vegan, and vegetarian diets, the international oat milk market reached US$ 4 billion in 2020 and is expected to grow with a compound annual growth rate of 9.8% by 2027 (49).

The presence of many substitutes like soya, whey, and others, which contain protein in heavy quantities and are demanded by the nutraceutical companies, lead to increased competition for oat protein products. In terms of food application, few oat protein products are available in the market given in Table 3, which urges food developers to build tailored strategies and food portfolios of these ingredients. As a functional food ingredient, oat protein application is still in the early stages, and food companies are investing in the production of high-quality oat protein products (44).

**TABLE 3:** Oat protein products available in the market.

Product name	Product type	Characteristics	Links
PrOatein Havreprotein	Oat proteins concentrate powder	Have mild taste of oat Suitable for cooking, baking and can be mixed with liquid or in smoothie. Contain no additive	https://www.apotea.se/proatein-havreprotein-450-g
Ideal oats^®^ Oat protein	Oat protein concentrate	Naturally contain all essential amino acids, including desirable BCAA’S, can help to boost up metabolism, muscle growth, tissue repair and may lower the cholesterol	https://www.idealoats.com/pages/plant-based-oat-protein-powder
Critical oats	Advanced protein porridge	Breakfast snack food supplement contains protein isolates. It can help to increase energy, improves digestion and nutrients absorption	https://appliednutrition.uk/products/critical-oats
Protein oat	Protein oat milk	Protein rich, creamy oat milk with 8 g of plant-based protein, calcium and vitamin D. Can be directly use as drink, pour over cereals, or add to smoothies.	https://www.califiafarms.com/products/original-protein-oat-milk-48oz
Muscle feast oats + isolates	Oats and whey protein powder	Natural, hormones and gluten free Oat and whey protein powder provide 31 g protein per serving. Protein isolates can be mixed with water or other beverages. Suitable for muscle growth and tissue repair.	https://www.musclefeast.com/products/oats-and-whey
Protein oats Oatmeal drink mix	Oat drink	Oatmeal drink mix can used in form of shake after workout to keep gut healthy, improve immunity and to faster muscle recovery	https://www.navafresh.com/products/borito-whey-protein-oats-belgian-chocolate
Oats and whey	Powder product	Oats and whey powder can be used with milk or water. It contains 35% of protein, contributes to the growth of muscle mass and also limits the catabolic process on muscles in muscle cells. Ideal to use before or after workout meal	https://ostrovit.com/en/products/ostrovit-oats-whey-1000-g-24313.html
Optimum nutrition oats and whey	Drinkable protein shake	Drinkable shake, easy to prepare and provide 24 g of protein per serving. Made up of whole oat flour and whey, free from artificial flavor, color and sweeteners. Contains fundamental elements for an athlete’s diet.	https://www.aasportsnutrition.com/product/optimum-nutrition-oats-and-whey/

## Nutraceutical Properties of Oat Protein-Derived Peptides

### Antioxidant Activity

Oats are known to contribute to a significant supply of antioxidants in the form of phytic acid, phenolic compounds, vitamin E, and avnanthramides to counter oxidative stress (50). The antioxidant defense system works to balance the reactive oxygen species by superoxide dismutase (SOD), glutathione peroxidase (GPx), catalase (CAT), vitamins, minerals, and co-factors to prevent cellular oxidative stress. These antioxidant compounds demonstrated radical scavenging activities by participating in a single electron transfer reaction (51). Oat proteins have also been considered a good source of antioxidant capacity, which have the potential to strengthen the treatment of oxidation-linked disorders, delay the oxidation process in foods and improve quality of life (52). Oat protein isolates and hydrolysates exhibited excellent antioxidant activities when assessed for 2,2 Azino-Bis-Ethylbenzoline-6-Sulfonic acid (ABTS), (HO**.**), (O**._2_**), and Fe(2**^+^**) chelating assays (53). Bioactive peptides (IRIPIL, FLKPMT, FNDILRRGQLL, LIGRPIIY, and NSKNFPTL) isolated from globulin fraction had the strongest 2,2-diphenyl-1-picryl-hydrazyl-hydrate (DPPH) (IC_50_ = 4.11 ± 0.07 mg/mL) and hydroxyl (IC_50_ = 1.83 ± 0.03 mg/mL) radical scavenging activity (54). In another study, oat peptides (GLVYIL, YHNAP, and GQTV) showed cytoprotective and peroxyl radical scavenging potential with considerable oxygen radical absorbance capacity (ORAC) value 0.67 ± 0.02, 0.61 ± 0.04, and 0.52 ± 0.01 Trolox equivalent (TE)/μM, respectively. The cytoprotective activity of peptides GLVYIL, YHNAP, and GQTV was correlated with the overall hydrophobicity of peptides, but not with their ORAC values (55).

Oat hydrolysates exhibited a cytoprotective effect by protecting HepG_2_ cells from AAPH-induced oxidative stress by significantly magnifying the activities of antioxidant enzymes, including GPx, CAT, and SOD, and inhibiting reactive oxygen species (ROS) levels (56). Furthermore, lipid oxidation and linolenic acid peroxidation were significantly inhibited up to 52, 35, and 16% with NINAHSVVY, YFDEQNEQFR, and SPFWNINAH peptides, respectively (57). The presence of tyrosine on (N or C-terminal) and histidine in a sequence of peptides improved their activity, as both these amino acids have good electron or proton donating ability. Hydrophobicity is also an important factor to interact with lipids (57). A recent development to alleviate anemia and oxidative stress has been made by using oat antioxidant peptide as a carrier to synthesize a novel oat peptide-ferrous (OP-Fe^+2^) chelate. OP-Fe^+2^ has shown to be helpful to alleviate anemia and also improved the activities of antioxidant enzymes, including GSH and SOD, while limiting the malondialdehyde (MDA) content in the liver of iron-deficient anemic rats model (58). In addition, oat peptides effectively recovered the H_2_O_2_ induced apoptosis and oxidative stress in human dermal fibroblast by regulating cellular antioxidant enzymes (59). Collectively, these reports demonstrated that oat-derived peptides can reduce oxidative stress and related disorders and may contribute to the development of nutraceutical or functional foods.

### Antidiabetic Activity

**FIGURE 2:**
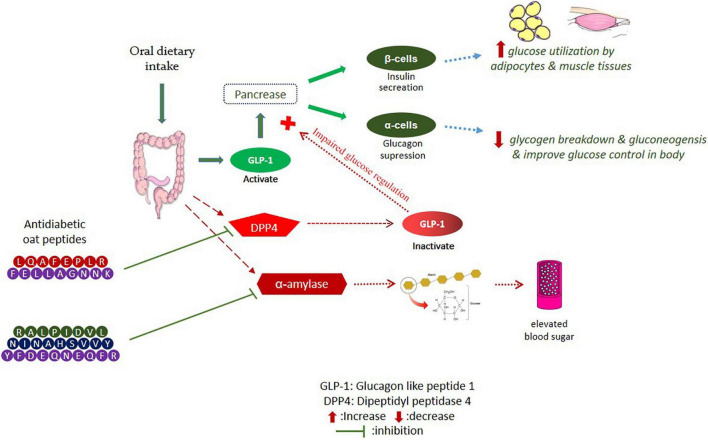
Inhibition of DPP4 and α-amylase pathways by oat bioactive peptides.

Oat peptides have the potential to inhibit dipeptidyl peptidase-4 (DPP4) and α-amylase and enhance the release of glucagon-like peptide-1 (GLP-1), as shown in Figure 2. Numerous dietary bioactive peptides work as antidiabetic agents by regulating insulin resistance and energy metabolism (60). Oat globulin peptides exhibited DPP4 inhibitory activity *in vitro*, with half-maximal inhibitory concentration (IC_50_) values of DPP4 at 100.4 μg/mL for peptide LQAFEPLR and 2.04 mg/mL for oat tryptic hydrolysates (61). Oat peptides showed improved DPP4 inhibition than fish peptides (42.5% at concentration of 5 mg/ml) and milk peptides IC_50_ = 0.60–2.14 mg/mL (55, 57, 62). Oat peptides inhibited the activity of DPP4 through binding to its active site with low binding energy and also downregulated the protein expression in Caco-2 cells. In addition, oat globulin peptides suppressed the α-glucosidase with IC_50_ of 35.67 μg/mL for LQAFEPLR and 113.8 μg/mL for hydrolysates (61). Walters et al. (63) have evaluated the antidiabetic potential of oat hydrolysates in NCI-H716 cells. The results indicate a significant inhibition of DPP4 (30.6–53.6%) and α-amylase (18–32%), and also improved insulin secretion, as well as glucose digestion. The inhibition mechanism has not been clarified, and a previous review (64) has shown that the inhibition of α-amylase by food-derived peptides usually occur through competitive binding between polysaccharide and peptides mainly through their aromatic amino acids. Another factor GLP-1 contributes to the stimulation of insulin, inhibition of glucagon by acting as an incretin hormone, and limits the postprandial glucose level. The secretion of glucagon-like peptide (GLP-1) was also improved in NCI-H716 cells, which ranges from 20.85 to 39.25 pM (picomole) (63). It has been shown that food-derived peptides affect gene expression, bile acid activation, and calcium receptors (65). Hence, peptidomics profile of hydrolysates is necessary to confirm their potential contribution to activity.

In another study, the peptides YFDEQNEQFR, NINAHSVVY, and RALPIDVL potentially inhibited α-amylase *in vitro*, with IC_50_ values of 37.5, 67.3, and 72.8 μM, respectively. The most active α-amylase inhibitory peptide was (YFDEQNEQFR) with acidic properties and containing tyrosine (Y) and arginine (R) amino acids, which may have contributed to the highest activity (57). Furthermore, oat peptides have been shown to effectively improve the symptoms of polydipsia, weight loss, and polyphagia, which can control the blood glucose level by improving insulin sensitivity and promoting glycogen synthesis (66). Oat grains and their bioactive compounds have been extensively studied for antidiabetic significance in human subjects. Regular oats intake for 23 weeks can improve fasting blood glucose level and insulin concentration by up to 20%, meanwhile, beta-glucans supplementation improves the blood glucose, insulin GLP-1, HbA1c, and appetite-regulating hormones in type 2 diabetic individuals (67, 68). However, work on the antidiabetic potential of oat peptides is still limited to *in vitro* studies; thus, systematic *in vivo* research is needed to fully understand the molecular mechanism behind the antidiabetic properties of oat bioactive peptides.

### Antihypertensive Activity

**FIGURE 3:**
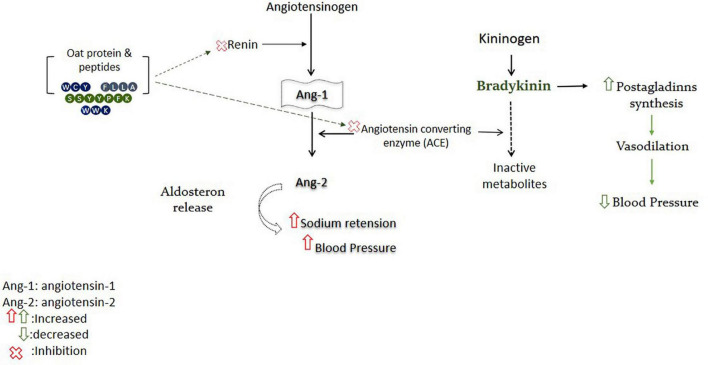
Oat-derived bioactive peptides improve blood pressure by targeting RAS.

Oat’s bioactive peptides have been shown to exert antihypertensive activity by targeting the renin–angiotensin system (RAS) (Figure 3). Oat protein isolates exhibited significant renin inhibitory activity, ranging from 40.50 to 70.5%, while the synthesized peptides showed comparatively lower inhibition than protein isolates. The attributes of renin inhibitory peptides are not clearly defined like ACE inhibitory peptides (69). Among the peptides, only IFFFL took part in renin inhibition due to the presence of bulky amino acids on the N-terminal, while the higher renin inhibitory activity of protein isolates could also be due to the presence of other bioactive compounds in protein, such as phenolic compounds (70). Oat protein isolates and peptides significantly inhibited the ACE, and protein isolates were found to inhibit ACE between 86.6 and 96.5%. The highest ACE inhibition value of protein is comparable with the positive control (captopril), which was found to inhibit the ACE by 97.7%. The synthesized peptides WCY, FLLA, and WWK were recorded for their highest ACE inhibition by 97.8, 97, and 95.3%, respectively. Peptide FEPL showed the lowest ACE inhibition value of 48.9% (70), and the lower ACE inhibition capacity of FEPL was due to the presence of proline, which has been suggested to reduce the binding affinity of the peptide with angiotensin-converting enzyme (ACE) (71).

Oat globulin peptide (SSYYPFK) was found to lower the systolic and diastolic blood pressure in a hypertensive rat model and exhibited high ACE inhibitory activity (IC_50_: 91.82μM). In addition, peptide SSYYPFK inhibited the renin by 28.6–45.59% and suppressed the production of intracellular endotheline-1 (ET-1) by 13.19–27.88% (72). Overexpression of ET-1 is an endogenous mediator of cardiovascular problems, such as hypertension or atherosclerosis. ET-1 and nitric oxide are important systems involved in the regulation of blood pressure, and peptide SSYYPFK may play an antihypertensive role by affecting these systems (73). Some clinical studies have been conducted on the antihypertensive effects of oatmeal, beta-glucan, dietary fibers, avenanthramides (AVA), and their fortified products. Most of these human interventional studies assessed the advantageous effects of oats and its derived active compounds in hypertensive and normotensive subjects by regulating systolic or diastolic blood pressure (74). Above mentioned research outcomes have confirmed that oat protein-derived peptides could effectively inhibit ACE and renin by targeting respective pathways and may be used as a functional ingredient for the prevention of hypertension and related disorders after interventional trials.

### Immunomodulatory Effects

Oat bioactive peptides exhibited immunomodulatory activity by improving innate and adaptive immunity. Oat oligopeptides (OOPs) attributed to the significant enhancement in interleukin IL, serum interferon (IFN)- γ, tumor necrosis factor (TNF)-α, T and Th cells percentage, immunoglobulin IgA, IgG production, as well as granulocyte macrophages colony-stimulating factors (GM-CSF) secretions (75). It also found that after OOP’s treatment, the percentage of CD3^+^ and CD4^+^ were enhanced, indicating the improvement in T cells quantity, which results in cytokines secretions and mediated cellular immune response (75). Specifically, CD4-T cells consists of T helper-1 (Th1) cells, which induce a cell-mediated immune response by producing IL-2, TNF-α, INF- γ, T-helper 2 (Th2), and GM-CSF to induce humorous responses by secreting IL-4, IL-5, and IL-10 cytokines (76). A previous review has shown that oat gluten protein and peptides can significantly enhance the ability of ConA-induced splenic lymphocyte transformation and delayed-type allergy. Meanwhile, a combination of oat peptides with American ginseng peptides has been shown to significantly improved the mice’s immunity (77). Hence, oat bioactive peptides are a potential source to improve immunity; however, the exact mechanism through which food-derived bioactive peptides regulate the immune system remains unclear.

### Antihypoxic Effects

Bioactive peptides from plant and animal sources have received much attention for their potential role in preventing hypoxia and other metabolic disorders (78–80). Oat oligopeptides (OOPs) can effectively ameliorate Hb, RBC, and Hct levels, which improves the oxygen-carrying capacity, as well as oxygen utilization rate of blood (81). The oxygen-carrying capacity of blood is directly reflected by the number of red blood cells and (HCT) indicating the volume ratio of RBCs to whole blood. Oxygen is attached to an iron atom and transported to the whole blood, and hemoglobin (Hb) combines with oxygen to transfer it from higher content areas to lower one’s, where it is needed (74, 77).

Oat oligopeptides also suppressed lactate and increased the activity of LDH, consequently upsurging the ability of the brain against lactic acidosis. The brain is more susceptible to oxidation because of its lipid membrane and weak antioxidant system. In the case of hypoxia, mitochondrial oxidase cannot completely reduce oxygen to water, resulting in the accumulation of reduced equivalent in the respiratory chain, which ultimately produces ROS. These factors trigger the lipid peroxidation in the brain and consequently, alkanes, epoxy fatty acids, alkanals, alkenals, and aldehyde, such as MDA, are produced (82–85). OOPs have been shown to decrease the MDA content of the brain, which minimizes lipid peroxidation and promotes angiogenesis, ultimately improving the hypoxic response (81).

### Antifatigue Activity

Oat bioactive peptides can alleviate the exercise-induced fatigue by improving muscle strength (86). Lactic acid accumulation and acidosis are widely considered to cause an increase in hydrogen ion concentration, which leads to a decreased action potential, inhibited sarcoplasmic reticulum uptake, and the release of calcium ions (87). Oat peptides effectively upregulated lactate dehydrogenase and suppressed the production of lactic acid. In addition, oat peptides downregulated the blood urea nitrogen and increased the level of glycogen in the liver and muscles (86). In another study, oat protein isolates have shown to significantly improve the physiological condition of mice by increasing the level of liver glycogen up to (19.64%), enhancing the activities of superoxide dismutase SOD by 20.27% in blood and 81.32% in muscles, and lactic dehydrogenase LDH (13.58%), and decreasing the level of malondialdehyde MDA by 3.45% in blood and 53.12% in muscles, and blood urea nitrogen BUN by 18.25% (88). Normally, hepatic glycogen plays an important role to complement blood glucose consumption and maintain its level in the physiological range. In the case of intensive exercise, glycogen depletion severely limits energy supply and maximal power output. Consequently, fatigue may happen, when most of the glycogen is consumed from the liver (89, 90). Urea is formed as the end product of protein metabolism in the liver, and blood urea nitrogen (BUN) is another sensitive parameter of fatigue, which dramatically increases after intensive exercise (91–93). Furthermore, decreased level of lactate dehydrogenase (LDH) is a biomarker of muscle damage. It is an enzyme of the glycolytic pathway and is dependent on NAD^+^ for the interconversion of pyruvate and lactate (94).

A clinical study confirmed that the supplementation of oat protein for 14 days prior and 4 post-exercise days alleviated the exercise-induced fatigue by preventing physical discomfort and reducing the elevation of plasma biomarkers, including creatine kinase, IL-6, C-reactive protein, and myoglobin content. In addition, oat protein prevented the decline of joint range of motion, jump performance, and muscle strength, and enhanced the post-exercise recovery of damaged muscles in healthy adults (95). These scientific studies depicted that oat protein can effectively alleviate exercise-induced fatigue.

### Antithrombotic Activity

The arachidonic acid (AA) pathway plays an important role in the platelet’s aggregation, AA directly acts on the COX1–TXA_2_ synthase pathway to produce TXA_2_. COX1 is a key enzyme in this pathway, associated with the metabolism of AA to induce TXA_2_ formation (96, 97). Finally, TXA_2_ causes changes in the shape of the platelet and activates fibrinogen receptors, consequently leading to platelet aggregation (98, 99). Hydrolysates produced from oats, barley, and buckwheat have been reported to have a crucial role in inhibiting platelet aggregation. Among all, oat hydrolysates showed high antiplatelet activity with an IC_50_ value of 0.282 mg/mL (100). Furthermore, oat globulin (small, mid, and large sized) fractions significantly inhibited the AA-induced platelet aggregation by (67.69, 17.98, and 14.33%) and increased the inhibitory rate to 73.11, 75.37, and 69.23%, respectively. These values are very close to the inhibitory rate of ASA (88.3%), which is a medical standard. The small-sized oat fraction showed more activity as compared to mid and large-size fractions (100). These results show that the inhibition of platelet aggregation by oat-derived bioactive peptides/hydrolysates could affectively act on COX1-TXA_2_ synthase pathway and may be employed to manage thrombosis and related chronic disorders.

### Hypocholesterolemic Effects

The cholesterol-lowering activity of oats is often attributed to its ability to reduce cholesterol absorption in the intestine or inhibit the enterohepatic circulation of bile acids by increasing the carriage of cholesterol and bile acid in the colon to facilitate their excretions through the feces. Oat contains numerous bioactive compounds to regulate cholesterol metabolism in animals (101). Hypocholestrolaemic activity of oat is strongly dependent upon their protein composition. A related study depicted a significant reduction in liver and plasma cholesterol levels of rats fed with hypercholestrolaemic diet by increasing fecal bile acids secretions (102). Oat protein significantly reduced the plasma low-density lipoprotein (LDL-C) and total cholesterol level in the liver by increasing the excretion of bile acids and regulating the liver CYP7A1 activity in an animal model (103). Regulation of specific genes and enzymes, such as LDLR, HMG CoA reductase, HYP3A4, and CYP7A1, is mainly involved in cholesterol homeostasis by converting cholesterol to bile acids or transporting it to hepatic cells and in bile acids metabolism (104).

A clinical study exhibited a significant reduction in triglycerides and total cholesterol of 268 hypercholesterolemic adults after consuming whole oat gain and β-glucan fortified products (105). Oat bran containing β-glucan was recorded for its remarkable effects in hyperchlesterolemic individuals and lowered the cholesterol by 23% without affecting the level of high-density lipoprotein HDL in the blood (106). Similarly, another clinical study claimed that the consumption of 6 g oat β-glucan for eight consecutive weeks effectively reduced the plasma LDL and total cholesterol (from 167.9 to 120.9 mg/dL and 231.8 to 194.2 mg/dL, respectively) and increased the plasma HDL from 39.4 to 49.5 mg/dL in overweight individuals with mild hypercholesterolemia (107). However, limited studies have been found on the hypocholestrolemic effects of oat protein and peptides.

## Processing and Purification of Bioactive Peptides

### Processing of Bioactive Peptides

Bioactive peptides are defined as specific fragments of proteins that have positive effects on body functions and improve human health. The composition and sequence of amino acids determine the activity of peptides when they are released from a precursor protein, where they are encrypted (108). Generally, bioactive peptides are produced by microbial fermentation or by the enzymatic hydrolysis of protein. Several microorganisms, including *Streptococcus salivarius* ssp. *thermophilus, Lactobacillus helveticus, Lactococcus lactis* ssp. *diacetylactis, Lactobacillus delbrueckii* ssp. *Bulgaricus*, and *Lactococcus lactis* ssp. *cremoris*, have been reported for their effective action to produce bioactive peptides or hydrolysates from natural food sources (109). In addition to live microbes, lactic acid-bacteria-derived proteolytic enzymes have also been successfully used for protein hydrolysis to produce bioactive peptides. Although microbial fermentation is mainly relevant to the production of peptides from dairy products, it has been shown that fermentation can also produce bioactive peptides from beans, wheat, rice, and soy (110–113).

**FIGURE 4:**
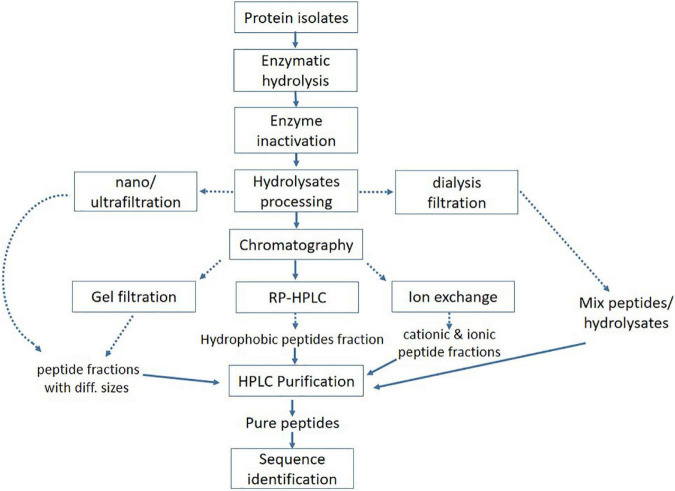
Conventional approach for processing and purification of bioactive peptides.

Enzymatic hydrolysis is a more efficient, safe, and reliable method than microbial fermentation as it takes less reaction time and is easy to control; additionally, it can be used to improve the biological and functional properties of proteins (114). These enzymes catalyze the hydrolysis of peptides bonds and may act on ester or amide bonds. All proteases have a certain degree of specificity in their substrate, generally based on a sequence of amino acids directly surrounding the bond that is cleaved (115). An extensive variety of bioactive hydrolysates and peptides have been produced from peanut, corn, soy, whey, and other protein sources (116–119). A conventional approach is mostly used to produce and identify the bioactive peptides (Figure 4). The efficacy of hydrolysates or peptides strongly depends upon the protein source, pretreatment of protein, proteases, and other hydrolysis factors, such as time, pH, and temperature (120). Proteases mainly involve two groups of enzymes i.e., exopeptidase (acts on amino or carboxyl ends of protein or peptides) and endopeptidase (acts on the interior of protein sequence) to break the peptide bonds (121). Most of the commercially available enzymes used for the production of bioactive peptides are derived from animals (e.g., pepsin or trypsin), microbial (e.g., Alcalase, Flavourzyme, and Neutrase), and plants (bromelain and papain) origin (122). Apart from commercial enzymes, some studies reported the crude enzymes for protein hydrolysis, suggesting the potential application of novel proteases source to produce bioactive peptides.

Several physical techniques, such as ultrasound, microwave, and high pressure, have been reported to show favorable effects on increased hydrolysis and release of potent bioactive peptides from precursor protein (123). Utilizing these cell disruptive green extraction techniques has proved to be more effective in protein recovery with minimum environmental pollution and also improves the yield, functional, and nutritional properties of proteins (124). The Ultrasonic waves can disrupt the food matrix and facilitate the extraction of protein (125). Ultrasound treatment can also affect the secondary structure of proteins, which can alter their behavior during enzymatic hydrolysis and consequently, improve the biological activities of hydrolysates. Numerous studies have shown the favorable effects of ultrasound on protein isolates of oat, corn, sunflower, rapeseed, and whey protein, as it helped in producing more active hydrolysates and short peptides, as well as improved the physicochemical and functional properties of protein isolates (63, 126–129). The microwave was shown to aid in chia seed proteolysis with improved bioactivity (antioxidant activity) and functionality (foaming and emulsifying properties) in a shorter time than simple enzymatic hydrolysis (130). Similarly, a pulsed electric field can enhance the antioxidant capacity of pine nuts by changing the secondary and tertiary structure of pentapeptide and protein (131). In addition to the above-mentioned isolation methods, chemical synthesis has also been used to obtain antioxidant, DPP4, and ACE inhibitory bioactive peptides (47, 64, 62). Most of the oat protein-derived bioactive peptides and hydrolysates discussed in this review have been produced using microbial and plant-based enzymes, including Alcalase, flavourzyme, and papain, while some peptides are produced by using gastrointestinal enzymes (trypsin and pepsin) as they mimic normal human digestion (48, 56, 94). However, enzyme–substrate ratio, degree of hydrolysis, and hydrolysis time are the important factors that need to be considered during the enzymatic hydrolysis process.

### Purification and Identification of Bioactive Peptides

Enzymatic hydrolysates need appropriate separation and purification to evaluate the structure–activity relationship as it contains a mixture of several bioactive peptides, unhydrolyzed protein, and polypeptides of different lengths among others. To evaluate the accurate structure–activity relationship, various separation and purification techniques, including membrane separation, size-exclusion chromatography, HPLC, UPLC, and RP-HPLC, are widely used to get purified bioactive peptides (132). Ultra-high pressure chromatography (UPLC) is most suitable to purify the small-sized bioactive peptides. The main advantages of UPLC include increased resolution, throughput, and sensitivity (133). Reverse-phase chromatography (RP-HPLC) is used to separate the peptides based on hydrophobicity (134). The hydrophilic interaction liquid chromatography (HILIC) is a useful technique to separate the hydrophilic peptides (135). In addition, HILIC is a valuable tool to improve the separation of short peptides and the differentiation of homologous sequence peptides through mass spectrometry (136). Membrane separations or ultrafiltration and gel electrophoresis have also been used as subsidiary approaches for the chemical or structural configuration of peptides (132, 137). Similarly, electrodialysis with filtration membrane (EDFM) could fractionate the active peptides from complex hydrolysates on a molecular charge and mass basis (138, 139). After a series of isolation and purification procedures, the peptide’s structure, and composition need to be identified. It is worth noting that mass spectrometry has greatly improved the process of studying protein profiles or hydrolysis products and identifying peptides’ sequence. Liquid chromatography mass-spectrometry (LC-MS) is most commonly used in the identification of peptides sequence due to the advantages of high efficacy, sensitivity, and good reproducibility (140, 141).

Peptides with antioxidant activity that were obtained from oat globulin by using alcalase were initially separated and purified with chromatography, and the most active fraction was applied to ESI-MS/MS to identify the sequence as FNDILRRGQLL, IRIPL, FLKPNIT, NSKNFPTL, and LIGRPIIY (54). Similarly, antidiabetic peptides that were also obtained from the hydrolysis of oat globulin with trypsin were passed through an ultrafiltration membrane and further purified on gel chromatography, and the peptides elute was applied to Nano-LC-ESI-MS/MS for sequence identification. Two highly active antidiabetic peptides were identified as LQAFEPLR and EFLLAGNNK (61). Although ultra-filtration can significantly increase the bioactivity of peptides, this technique is not enough to get a highly pure product. Besides, the membrane is easily blocked by raw material, which causes pollution and the waste of raw material. Different techniques have some advantages and disadvantages, which make it difficult to obtain ideal peptides by using only a single technology. Therefore, the combination of separation techniques can attain precise classification and separation of peptides mixture to obtain high pure peptides.

## Conclusion and Recommendation

In summary, oats protein with higher content, unique amino acid composition, and less environmental impact in sense of land use, GHG emissions, and carbon footprint is a potential candidate for developing plant protein-based functional products. Oat protein with no allergic characteristics is likely to play a significant role in the meat alternative market over pea and soy proteins. It can be used to incorporate as a functional ingredient in various food products or to produce improved quality yogurt. Besides, the health benefits of oat protein and peptides make these compounds nutraceutical food additives in the formulation of functional foods. Enzymatic hydrolysis is a more efficient and reliable method to produce active peptides. Oat peptides have been shown to have remarkable biological activities by targeting specific molecular pathways of various chronic disorders. These peptides are considered health improving and disease-preventing agents by functioning as an antidiabetic, antihypertensive, antioxidant, anti-hypoxia, anti-fatigue, antithrombotic, and anti-hypercholesterolemia among others. However, more research should be carried out to evaluate the bioavailability and interactions of oat peptides with other food components and body organs to determine their wellbeing for human consumption. It is also important to develop and implement strategies to confirm the valorization of the nutritional and functional potential of oat protein and peptides for their exploration in the development of marketable nutraceutical and functional foods.

## Author Contributions

HR collected the data and drafted the manuscript. RD and XW helped to design the study. AA constructed the figures. RA, LZ, and LL revised the manuscript. XH supervised, revised, and approved the final version for publication. All authors contributed to the article and approved the submitted version.

## Conflict of Interest

LL was employed by Guilin Seamild Food Co., Ltd. The remaining authors declare that the research was conducted in the absence of a commercial or financial relationship that could be construed as a potential conflict of interest.

## Publisher’s Note

All claims expressed in this article are solely those of the authors and do not necessarily represent those of their affiliated organizations, or those of the publisher, the editors and the reviewers. Any product that may be evaluated in this article, or claim that may be made by its manufacturer, is not guaranteed or endorsed by the publisher.
